# Psychometric properties of the Flemish translation of the NEECHAM Confusion Scale

**DOI:** 10.1186/1471-244X-5-16

**Published:** 2005-03-25

**Authors:** Koen Milisen, Marquis D Foreman, Annik Hendrickx, Jan Godderis, Ivo L Abraham, Paul LO Broos, Sabina De Geest

**Affiliations:** 1 Center for Health Services and Nursing Research, Katholieke Universiteit Leuven, Leuven, Belgium & Department of Geriatrics, University Hospitals Leuven, Leuven, Belgium; 2 Department of Medical-Surgical Nursing, College of Nursing, University of Illinois at Chicago, Chicago, IL, USA; 3 Covance Clinical and Periapproval Services SA, Brussels, Belgium; 4 Department of Psychiatry, Faculty of Medicine, Katholieke Universiteit Leuven, Belgium; 5 University of Pennsylvania School of Nursing & Leonard Davis Institute of Health Economics, Wharton School of Business, Philadelphia, PA, USA and Matrix45, Earlysville, VA, USA; 6 Department of Surgery, University Hospitals, Katholieke Universiteit Leuven, Leuven, Belgium; 7 Institute of Nursing Science, University of Basel & Division of Clinical Nursing Science, University Hospital of Basel, Switzerland

## Abstract

**Background:**

Determination of a patient's cognitive status by use of a valid and reliable screening instrument is of major importance as early recognition and accurate diagnosis of delirium is necessary for effective management. This study determined the reliability, validity and diagnostic value of the Flemish translation of the NEECHAM Confusion Scale.

**Methods:**

A sample of 54 elderly hip fracture patients with a mean age of 80.9 years (SD = 7.85) were included. To test the psychometric properties of the NEECHAM Confusion Scale, performance on the NEECHAM was compared to the Confusion Assessment Method (CAM) and the Mini-Mental State Examination (MMSE), by using aggregated data based on 5 data collection measurement points (repeated measures). The CAM and MMSE served as gold standards.

**Results:**

The alpha coefficient for the total NEECHAM score was high (0.88). Principal components analysis yielded a two-component solution accounting for 70.8% of the total variance. High correlations were found between the total NEECHAM scores and total MMSE (0.75) and total CAM severity scores (-0.73), respectively. Diagnostic values using the CAM algorithm as gold standard showed 76.9% sensitivity, 64.6% specificity, 13.5% positive and 97.5% negative predictive values, respectively.

**Conclusion:**

This validation of the Flemish version of the NEECHAM Confusion Scale adds to previous evidence suggesting that this scale holds promise as a valuable screening instrument for delirium in clinical practice. Further validation studies in diverse clinical populations; however, are needed.

## Background

Delirium, as defined by the Diagnostic and Statistical Manual of Mental Disorders, Fourth Edition, text revised (DSM-IV-TR), is a disturbance of consciousness with reduced ability to focus, sustain or shift attention, a change in cognition or the development of a perceptual disturbance that occurs over a short period of time and tends to fluctuate over the course of the day [1]. This transient and etiologically non-specific, organic cerebral syndrome of acute onset, is a common and serious health problem for elderly hospitalized patients [2-9]. Several studies have shown that patients who develop delirium are at increased risk for morbidity, longer and costlier hospitalizations, nursing home placement and death [9-11]. Because of significant and profound negative outcomes of delirium, and its potential reversibility, prompt and effective treatment is dependent upon the early recognition of delirium [12-14]. However, nurses and physicians have been shown to consistently underdiagnosis delirium [12,14-16]. The underrecognition and misdiagnosis of delirium is partly a function of the failure of providers to consistently use standardized methods of detection [15].

Several standardized instruments for the routine, systematic, and comprehensive assessment of changes in cognitive status or detecting delirium have been described in greater detail elsewhere [17-20]. The most common method for assessing cognition is the mental status questionnaire, with the most frequently used bedside test of cognition being Folstein's Mini-Mental State Examination [21]. However, the usefulness of mental status questionnaires is equivocal since they pose significant response and administration burdens for patient and nurse [22], especially when repeated administration is needed for monitoring or following the pattern of a delirium episode, thereby limiting their clinical usefulness. Additionally, the interpretation of the resulting score is not always clear and can be misleading, as the severity of the cognitive impairment, level of formal education, fatigue, and characteristics of the testing environment adversely influence performance on mental status testing [23]. Moreover, nurses favor instruments that are more heavily based on the observation of behavior than formal testing, those that can be used in the course of usual practice, repetitively and without respondent burden [24].

In response to this criticism, Neelon and colleagues [22,25] developed the NEECHAM Confusion Scale. The advantages of the NEECHAM over standard cognitive testing are that the NEECHAM can be rapidly completed (during 10 minutes) by the nurse at the bedside using a structured database derived during routine nursing assessments and interactions with patients. Because the NEECHAM places a minimal response burden on the patient, and because it is comprised of items that have no learning effect, testing can be repeated at frequent intervals to monitor changes in the patient's cognitive status. This can be of major importance, since symptoms of a delirium vary in intensity and fluctuate diurnally [1,13]. Additional advantages of the NEECHAM are that it can detect delirium in its early stage, and is sensitive to both the hyperalert-hyperactive and hypoalert-hypoactive variants of delirium [22].

Several studies have reported acceptable psychometric properties of the original English version [22,25-27] and of a Swedish translation [28] of the NEECHAM Confusion Scale (Table 1). The aim of the current study was to determine the reliability, validity and diagnostic value of the Flemish translation of the NEECHAM Confusion Scale.

**Table 1:** Overview of psychometric properties of the NEECHAM Confusion Scale

Author	Sample	Reliability	Validity	Diagnostic Values
Champagne et al. [26]	n = 35 (21 hospitalized and 14 nursing home patients) age ≥ 69 years	Internal consistency = 0.85 Interrater reliability = 0.96 Test-retest reliability = 0.98	Concurrent: Correlation with MMSE = 0.81 Construct: Factor analysis revealed 2 factors (% of variance explained not given)	
Neelon et al. [25]	n = 158 medical patients age ≥ 65 years			At least two of three clinical indicators (MSP^1^, MMSE^2^, DSM-III^3^) = reference criterion; and NEECHAM cut-off = 24/25: sensitivity: 95% specificity: 78% predictive value: 57% false positive: 17% false negative: 3%
Neelon et al. [22]	Sample 1: n = 168 medical patients age ≥ 65 years	Sample 1: Internal consistency = 0.90	Sample 1: Concurrent: * Correlation with MMSE = 0.87 * Correlation with DSM-III-R total score = -0.91 * Correlation with diagnosis of delirium by DSM-III-R diagnostic criteria = -0.70 Construct: Factor analysis revealed 2 factors (explaining 72% of variance)	
	Sample 2: n = 258 medical patients age ≥ 65 years	Sample 2: Internal consistency = 0.90	Sample 2: Concurrent: * Correlation with DSM-III-R total score = -0.86 * Correlation with diagnosis of delirium by DSM-III-R diagnostic criteria = -0.54 Construct: Factor analysis revealed 2 factors (% of variance explained not given)	
Csokasy [27]	n = 19 patients on intensive care unit age ≥ 65 years	Internal consistency = 0.81	Concurrent: * correlation with oxygen saturation below 91 = 0.93 * correlation with DSM-III-R total score = 0.68	
Johansson et al. [28]	n = 73 patients with hip fracture age ≥ 60 years	Before surgery: Internal consistency = 0.73 After surgery: Internal consistency = 0.82	Before surgery: Construct: Factor analysis revealed 2 factors (explaining 57.4% of variance) and 3 factors (explaining 69% of variance) After surgery: Construct: Factor analysis revealed 2 factors (explaining 61.5% of variance) and 3 factors (explaining 73.6% of variance)	

^1^MSP = chart report of mental status problem

^2^MMSE = Mini-Mental-State Examination

^3^DSM-III-R = Diagnostic and statistical manual of mental disorders, 3^rd ^ed., revised

## Methods

### Design and sample

This study is a secondary data analysis of the experimental group of a larger intervention study [29] that was approved by the local ethics committee of the University Hospitals of Leuven, Belgium. Subjects were recruited at the emergency room of the University Hospitals of Leuven (Belgium). Patients were eligible if they had a traumatic fracture of proximal femur (intra- and extra-capsular) and were hospitalized to one of the two traumatological nursing units within 24 hours after surgery (convenience sampling). Subjects had to be 65 years of age or older, Flemish-speaking, and had to give informed consent. Exclusion criteria were multiple trauma, concussion of the brain, pathological fractures, surgery occurring later than 72 hours after admission, aphasia, blindness, deafness and fewer than 9 years of formal education (the latter because of validity concerns of the MMSE with lesser educated subjects [23]).

A total of 60 older patients with a hip fracture were admitted to the study during a 5-month period. Six patients were excluded from analyses because of missing data for demographic variables and/or co-morbidity. From the remaining 54 patients, the majority was female (81.5%) with a mean age of 80.9 years (SD = 7.8) (Table 2).

**Table 2:** Demographic variables and co-morbidity (n = 54)

Mean age in years (SD)		80.9 (7.8)
		
Gender, n (%)	Male	10 (18.5)
	Female	44 (81.5)
		
Marital status, n (%)	Single	6 (11.1)
	Married	16 (29.6)
	Widowed	31 (57.4)
		
Type of hip fracture, n (%)	Intra-capsular	7 (13.0)
	Extra-capsular	47 (87.0)
		
Co-morbidities, n (%)	Dementia	7 (12.9)
	Depression	5 (9.3)
	Cardiac	8 (14.8)
	Pulmonary	8 (14.8)
	Hypertension	11 (20.4)
	Diabetes	9 (16.7)
	Other	15 (27.7)

### Instruments

The NEECHAM Confusion Scale consists of nine components organized in three subscales, allowing the detection not only of changes in cognitive status, but also changes and different patterns of physiological and behavioral manifestations over short time frames [22]. Subscale 1 (« Processing ») (0 to 14 points) focuses on the primary components of cognition: attention/alertness, verbal and motor command of information, and memory and orientation. Subscale 2 (« Behavior ») (0 to 10 points) measures behavioral manifestations associated with more physical performance functions: appearance/posture control, sensorimotor performance and verbal manifestations accompanying or heralding deliriumlike syndromes. Subscale 3 (0 to 6 points) consists of physiological control and stability, including vital functions, oxygenation, and continence. These last 3 components in subscale 3 are an attempt to link the cognitive and behavioral symptoms of delirium with alteration in various physiologic parameters to facilitate the identification of the underlying etiolog(y)(ies) [22,24]. The NEECHAM total score is calculated by adding the scores for the three subscales, and ranges from 0 (minimal responsiveness) to 30 (normal function) points. The following cut-points are suggested for clinical practice: 0 to 19 indicates acute and moderate confusion, to severe confusion and/or delirium, to nonresponsiveness; 20 to 24 indicates mild disturbance in information processing, to mild or early development of confusion and/or delirium; 25 to 26 indicates risk for confusion and/or delirium; and scores equal or greater than 27 indicate normal cognitive functioning or the absence of confusion and/or delirium [30,31].

The NEECHAM instrument was translated into Flemish by the first author (KM) and examined by the fourth co-author (JG). Both authors have good knowledge of English and wide experience in assessing forms for grading cognitive and behavioural state.

Delirium was assessed by using the English version of the Confusion Assessment Method (CAM) [32]. The CAM is an easy to use diagnostic algorithm to efficiently and effectively detect delirium that was developed from the DSM-III-R criteria for delirium [33]. The CAM consists of 9 diagnostic criteria of which 4 are considered core criteria for delirium: (1) acute onset and fluctuation, (2) inattention, (3) disorganized thinking, (4) altered level of consciousness. The first two and at least one of the other two criteria must be fulfilled to classify a patient as delirious. The diagnostic value of the CAM is excellent when using psychiatric diagnosis as the gold standard. More specifically, the CAM has a 94%–100% sensitivity, 90%–95% specificity, 91%–94% positive predictive value and 90%–100% negative predictive value [32]. Interobserver reliability (Kappa) ranged between 0.81–1.0. The CAM also was shown to have convergent agreement with other mental tests, including Mini-Mental State Examination (k = 0.64) [21], Visual Analog Scale for Confusion (k = 0.82) [34], Digit Span test (k = 0.66) [35,36] and a memory recall test (k = 0.59) [37].

To measure the severity of delirium, scoring of the CAM was modified in consultation with the originator of the instrument [29]. Six items of the CAM (inattention, disorganized thinking, disorientation, memory impairment, perceptual disturbances, and psychomotor agitation/retardation) were scored as 0, 1 or 2 points indicating absent, present in mild form or present in severe form, respectively, rather than merely present or absent. A seventh item (altered level of consciousness) was scored as alert (0 points), vigilant or lethargic (1 point), and stupor or coma (2 points). The total score ranges from 0 to 14, in which a higher score indicates greater severity of delirium.

Cognitive impairment was assessed using the Mini-Mental State Examination [21], an 11-item instrument measuring orientation, memory, attention, the ability to name, ability to follow verbal and written commands, ability to write a sentence spontaneously, and ability to copy a geometric figure. The total score is obtained by summing the correct responses, yielding a score between 0 to 30 (total score below 24 indicating cognitive impairment). The MMSE is a reliable and valid measurement instrument for screening and rating severity of cognitive functioning, reported to be psychometrically robust across settings, and despite gender, race, ethnicity, and social class [19,23].

### Procedures

All nurses of the emergency department and both traumatological units were trained by the first author (KM) in the use of the NEECHAM Confusion Scale. Using this instrument in the standard nursing care plans of older patients with hip fracture was part of a larger intervention program for delirium [29]. Patients were evaluated by the nurses using the NEECHAM within 12 hours of admission to the emergency department, and during the morning shift on the first, third, fifth and eighth postoperative days at the traumatological units. For measuring oxygen saturation and vital function (Subscale 3 of the NEECHAM), a noninvasive pulse-oximeter (NPB-40, Mallinckrodt Belgium NV) and standard clinical methods were used, respectively.

The MMSE and CAM were administered by 3 trained research nurses on the same measurement points of the NEECHAM evaluation.

### Statistical analyses

Fifty-four patients were each assessed at 5 data collection measurement points, generating a total of 270 observations. However, not all NEECHAM evaluations by the registered nurses could be used, because of incomplete data. Therefore, only 194 observations (30, 36, 40, 43 and 45 observations on measurement points 1 till 5, respectively) were eligible for statistical analysis. First, the distribution of the total NEECHAM scores and items were examined. Further, the internal consistency of the scale was tested by calculating Cronbach's alpha, item-total and inter-item correlations. To explore the inter-item correlations, principal components analysis was performed. Paired observations of the nurses and research nurses were used to test concurrent validity, meaning that total NEECHAM scores were correlated with total scores on the same measurement points on the modified version of the CAM and on the MMSE, respectively (Pearson correlation coefficient). To explore the diagnostic value of the NEECHAM scale to identify subjects as being delirious or not, a receiver operating characteristic curve (ROC) was constructed and sensitivity, specificity, positive and negative predictive values and accuracy were examined for all possible total NEECHAM scores (0–30) using the CAM diagnostic algorithm as gold standard.

## Results

### NEECHAM Scores and item distribution

The total score on the NEECHAM scale ranged from 6 to 30, with a mean of 26.7 (SD = 4.99). According to the NEECHAM classification a total score less than or equal to 19 was found for 17 observations (8.8%). Another 17 observations (8.8%) ranged between 20 and 24 and 25 observations (12.9%) ranged between 25 and 26. For all other observations (n = 135, 69.5%) scores were 27 or greater. Further, a full range of scores was observed for all items (Table 3), except for the item "command" processing for which an almost full range was observed (score 0 not observed), indicating good responsiveness for all items.

**Table 3:** Total NEECHAM scores and item distribution (n = 54 patients, 194 test occasions)

NEECHAM item	Mean	SD	Minimum score	Maximum score
Attention processing (score 0 to 4)	3.65	0.74	0	4
Command processing (score 0 to 5)	4.56	0.94	1	5
Orientation processing (score 0 to 5)	4.42	1.08	0	5
Appearance behavior (score 0 to 2)	1.77	0.49	0	2
Motor behavior (score 0 to 4)	3.62	0.90	0	4
Verbal behavior (score 0 to 4)	3.69	0.75	0	4
Vital function stability (score 0 to 2)	1.72	0.53	0	2
Oxygen saturation stability (score 0 to 2)	1.74	0.53	0	2
Urinary continence (score 0 to 2)	1.52	0.76	0	2

Total NEECHAM score (score 0 to 30)	26.69	4.99	6	30

### Reliability

Internal consistency of the scale was tested by calculating Cronbach's alpha, inter-item and corrected item-total correlations. The alpha coefficient for the total NEECHAM score was high (0.88) and increased by omitting the items 'vital' and 'oxygen' (Table 4).

**Table 4:** Pearson Item-total correlation coefficients of NEECHAM Confusion Scale (n= 54 patients, 194 test occasions)

NEECHAM item	Corrected item-total correlation	Total alpha if item is deleted
Attention	0.8148	0.8550
Command	0.8827	0.8448
Orientation	0.8579	0.8484
Appearance	0.7113	0.8701
Motor	0.8031	0.8537
Verbal	0.8689	0.8499
Vital	-0.0403	0.9091
Oxygen	0.1799	0.8982
Continence	0.5408	0.8777

For item-total correlations (Table 4), the processing (attention, command, orientation) and behavior (appearance, motor, verbal) items correlated strongly with the sum of the other items (Pearson r > 0.7113), while urinary continence and both vital function and oxygen saturation correlated modestly (Pearson r = 0.5408) and weakly (Pearson r < 0.1799), respectively. For the item vital function, the correlation was even negative.

Pearson inter-item correlation coefficients among the items ranged from 0.0049 to 0.8830 (Table 5). For oxygen saturation, low correlations were found between all other items (Pearson r from 0.0766 to 0.1995). Also vital function showed low and negative correlations with most of the other items (Pearson r from -0.0434 to -0.1329) except for the items appearance, oxygen and continence which were still low but positive (Pearson r = 0.0049, 0.1995, 0.0856, respectively). All other items correlated moderately to highly (Pearson r from 0.3791 to 0.8830).

**Table 5:** Pearson Inter-item correlation coefficients of NEECHAM Confusion Scale (n = 54 patients, 194 test occasions)

ITEMS	Attention	Command	Orientation	Appearance	Motor	Verbal	Vital	Oxygen	Continence
Attention	1.0000								
Command	0.8428	1.0000							
Orientation	0.7955	0.8421	1.0000						
Appearance	0.6190	0.7076	0.6670	1.0000					
Motor	0.6998	0.7811	0.7526	0.6631	1.0000				
Verbal	0.8155	0.8830	0.8223	0.6831	0.7805	1.0000			
Vital	-0.1329	-0.0950	-0.0893	0.0049	-0.0434	-0.0691	1.0000		
Oxygen	0.1274	0.1346	0.1725	0.0869	0.1815	0.1321	0.1995	1.0000	
Continence	0.4756	0.5056	0.5384	0.3791	0.4620	0.4748	0.0856	0.0766	1.0000

### Construct validity

To further explore the inter-item correlations, principal components analysis was performed. Two eigenvalues greater than 1 were found, suggesting that, at most, only 2 sources contributed substantially to the shared variance among the items. A two-component solution accounted for 70.8% of the total variance in the items, in which the first and second component explained 57.3% and 13.5%, respectively. The items attention, command, orientation, appearance, motor behavior, verbal behavior and urinary incontinence loaded fairly high (0.6105) to high (0.9411) on the first component (Table 6). Vital function and oxygen saturation showed relatively high correlations (> 0.7080) with the second component. Urinary incontinence had a crossloading of 0.1560 on component 2 and oxygen saturation had a crossloading of 0.1872 on component 1, both indicating some minor association with that respective dimension. The communalities, meaning the proportion of the variance of that variable explained by both components, ranged for most variables between 0.6228 and 0.8892. In other words, these variables had relatively high to high relation with the 2 components. Communalities for oxygenation and vital function were rather low, but still above commonly accepted lower limits (0.3971 and 0.5364, respectively). Orthogonal and oblique axis rotation did not improve the initial unrotated solution.

**Table 6:** NEECHAM Confusion Scale Component Loadings and Communalities (n = 54 patients, 194 test occasions)

	Loadings		
NEECHAM item	Component I	Component II	Communalities
Attention	0.9411	-0.0590	0.7991
Command	0.9246	-0.0430	0.8892
Orientation	0.9155	-0.0185	0.8385
Appearance	0.8886	-0.0967	0.6228
Motor	0.8682	0.0298	0.7546
Verbal	0.7891	0.0042	0.8568
Vital	-0.0715	0.8205	0.5364
Oxygen	0.1872	0.7081	0.3971
Continence	0.6105	0.1560	0.6794

### Concurrent validity

To test concurrent validity of the NEECHAM Confusion Scale, paired observations of total NEECHAM scores of the nurses were correlated with total MMSE scores and total CAM scores of the researchers, respectively. A strong positive correlation coefficient (r = 0.75) was found with the MMSE, indicating that higher MMSE scores (better neurocognitive functioning) were related to higher NEECHAM scores (less severe confusion and/or delirium). Also, a strong negative correlation coefficient (r = -0.73) was found with the CAM, indicating that higher total CAM scores (more severe delirium) were related to lower total NEECHAM scores (more severe confusion and/or delirium).

### Diagnostic values

**Figure 1:**
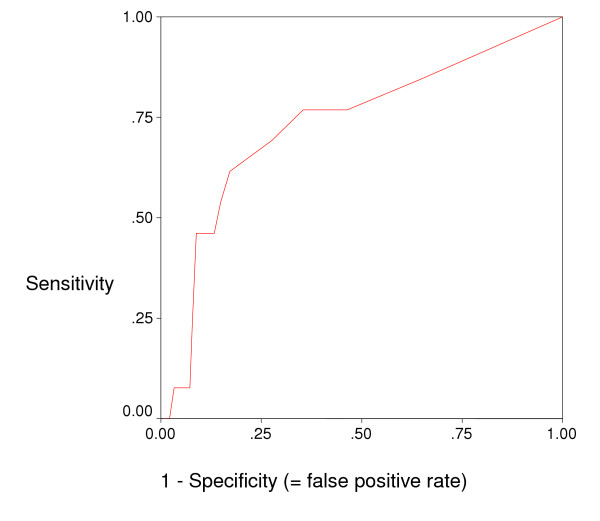
Receiver Operating Characteristic curve (ROC; Eng [41]).

A sensitivity analysis was performed by using ROC curve (Figure 1) and exploring sensitivities and specificities for different cut-offs of the total NEECHAM scores to determine the most optimal cut-off (table 7). ROC showed an area under the curve of 0.733 (Confidence Interval = 0.578–0.888). Based on ROC and diagnostic value of the different cut-offs, the most optimal cut-off point was found to be 27. This cut off showed a fairly good sensitivity of 76.9%, an acceptable specificity (64.6%) and a high negative predictive value (97.5%). However, the positive predictive value was low (13.5%), though the accuracy was acceptable (65.5%).

**Table 7:** Sensitivities and specificities for a subset of threshold values of the NEECHAM total score (n = 54 patients, 194 test occasions)

	CAM algorithm		
NEECHAM total score	Delirious N	Not delirious n	Total N

≤ 24	7	27	34
≥ 25	6	154	160
Total	13	181	194

Sensitivity [(7/13) × 100] = 53.8%; Specificity [(154/181) × 100] = 85.1%; Predictive value of a positive test [(7/34 × 100] = 20.6%; Predictive value of a negative test [(154/160) × 100] = 96.3%; Accuracy [(7+154/7+27+6+154) × 100] = 83.0%			

≤ 25	8	31	39
≥ 26	5	150	155
Total	13	181	194

Sensitivity = 61.5%; Specificity = 82.9%; Predictive value of a positive test = 20.5%; Predictive value of a negative test = 96.8%; Accuracy = 81.4%			

≤ 26	9	50	59
≥ 27	4	131	135
Total	13	181	194

Sensitivity = 69.2%; Specificity = 72.4%; Predictive value of a positive test = 15.2%; Predictive value of a negative test = 97.0%; Accuracy = 72.2%			

≤ 27	10	64	74
≥ 28	3	117	120
Total	13	181	194

Sensitivity = 76.9%; Specificity = 64.6%; Predictive value of a positive test = 13.5%; Predictive value of a negative test = 97.5%; Accuracy = 65.5%			

≤ 28	10	84	94
≥ 29	3	97	100
Total	13	181	194

Sensitivity = 76.9%; Specificity = 53.6%; Predictive value of a positive test = 10.6%; Predictive value of a negative test = 97.0%; Accuracy = 55.2%			

≤ 29	11	118	129
30	2	63	65
Total	13	181	194

Sensitivity = 84.6%; Specificity = 34.8%; Predictive value of a positive test = 8.5%; Predictive value of a negative test = 96.9%; Accuracy = 38.1%			

## Discussion

The purpose of this study was to determine the validity and diagnostic values of the Flemish translation of the NEECHAM Confusion Scale. In line with previous evidence, the findings of this study support the validity of the NEECHAM Confusion Scale (Table 1). Diagnostic values however were somewhat different than evidence from previous studies.

Reliability analyses showed a high internal consistency (alpha coefficient = 0.88). Only Neelon et al. [22], reported a higher internal consistency (0.90), with the same physiological items (vital function and oxygen saturation) showing low item-total and inter-item correlations. As a matter of fact, item-total correlation for vital function in this study was extremely low and even negative (-0.0430 compared to 0.30 and 0.32 in the study of Neelon et al. [22]). In comparison with our study, Neelon et al. [22] tested the scale in two samples (n= 168 and 258, respectively) and data were not based on repeated measurements.

Based on the low item-total and inter-item correlations of vital function and oxygen saturation, it could be suggested to delete these two items from the NEECHAM Scale. When we deleted the items 'vital' and 'oxygen' in our study, internal consistency of the NEECHAM increased as expected. Further evidence for this approach was our factor analyses which confirmed the findings of Neelon et al. [22] and Johansson et al. [28] showing that the vital function and oxygen items do not load on the general factor that includes the seven other items. The inclusion of such specific physiologic data seems to artificially constrain the scale's use [19] but needs to be further evaluated in future studies before excluding these items from the NEECHAM Scale.

In line with Neelon et al. [22] and Johansson et al. [28], the continence item correlated with all items of the original subscale 1 (processing) and 2 (behavior). The continence item also loaded fairly high on the first component in the factor analysis. These findings suggest that continence rather seems to be an important indicator for delirium than belonging to a 3^rd ^subscale (e.g. physiological changes).

Concurrent validity was good. The high correlations between the NEECHAM and the MMSE and CAM instruments (r = 0.75 and -0.73, respectively) – together with the variation observed in NEECHAM total scores and item distribution – indicate that the NEECHAM is a valuable instrument for rating severity of both cognitive dysfunctioning and delirium, respectively.

Although values for sensitivity (76.9%) and specificity (64.6%) in our study are lower than those reported by Neelon et al. [25] (Table 1), these values are still within acceptable ranges. The area under the ROC curve, indicating how well the test separates the group being tested into those with and without delirium, was found to be fairly good (= 0.773). Positive predictive value was however low (13.5%) indicating the need for further studies to optimize this aspect of the scale. It has however to be mentioned that the scale was successfully used by resource nurses in an intervention study as demonstrated by Milisen et al. [29].

We found an optimal cut-point of 27 (see table 7) for detection of delirium and this in contrast with Neelon et al. [22] who found an optimal cut-point of 24 (see table 1). However, whether our cut-point or Neelon's [22] cut-point is ideal for different hospital settings is not known. Therefore, future studies should include a sensitivity analysis to explore which cut-off point is most valuable to detect delirium for different in-hospital patient populations. Neelon's study focused on older patients hospitalized for acute medical illness whereas our study included older post surgical hip fracture patients. Further, rather than merely comparing single scores with a threshold value, identifying a patient at risk for delirium may also be addressed by examining an acute decrease in total NEECHAM scores over time (e.g. 2 or more points), as recently shown by Matsushita et al. [31].

Another aspect that deserves further consideration is the reference criteria used. Neelon et al. [25] included at least two of the following clinical indicators: chart report of mental status problem, low MMSE score and presence of DSM-III symptoms for delirium versus use of the CAM-criteria in the current study. When however compared to other cognitive screening instruments or criteria published so far, the CAM appears to have more favorable sensitivity and specificity for detection of delirium [38].

Limitations of our study are as follows. Generalization of the findings may be hampered by the sampling methods (secondary analysis of data collected for the experimental group of a non-randomized trial) and the sample (restriction to older patients with hip fracture). Therefore, further studies including patients presenting a broad range of ages and clinical conditions (e.g. surgical and non-surgical patients) would benefit the generalization of findings. Our analyses were based on 194 observations in 54 older patients. Admittedly, these observations were not independent which could have potentially influenced the results. However, the main objective of this study was descriptive, not inferential, and this is not expected to be seriously affected by non-independence [39,40]. Furthermore, our findings concur with previous published evidence on reliability, validity and diagnostics value of the scale. It also needs to be mentioned that low prevalence of delirium (13/194 = 6.7%) could be an exploratory factor for the low positive predictive value. The higher the disease prevalence the greater the probability that a positive result will be 'correct'. Studies in samples with various prevalences of delirium are therefore indicated to test robustness of the findings.

## Conclusion

This study contributes to the validation process of the (Flemish version of the) NEECHAM Confusion Scale. In line with previous evidence, this instrument seems to be a promising tool for clinical practice to screen for delirium in older hospitalized patients and to monitor for the course of cognitive dysfunction and severity of delirium. The minimal response burden on patients, even when used repeatedly, is certainly also an asset in clinical settings. Further validation studies (preferably not using added observations) in diverse clinical populations are however needed.

## Competing interests

The author(s) declare that they have no competing interests.

## Authors' contributions

KM, MDF, IAL, PLOB proposed the original idea for the study and designed the study protocol. KM and JG contributed to the translation part of the study. KM and AH oversaw field activity and executed statistical analyses. KM, MDF, AH, JG, IAL, PLOB, and SDG were involved in the interpretation of data. KM drafted the manuscript. MDF and SDG revised the manuscript critically for important intellectual content. All authors read and approved the final manuscript.

## Pre-publication history

The pre-publication history for this paper can be accessed here:


